# Skeletal Editing: Ring Insertion for Direct Access to Heterocycles

**DOI:** 10.3390/molecules29091920

**Published:** 2024-04-23

**Authors:** Xue Li, Zhigang Xu

**Affiliations:** College of Pharmacy, National & Local Joint Engineering Research Center of Targeted and Innovative Therapeutics, IATTI, Chongqing University of Arts and Sciences, Chongqing 402160, China; lxshirley@cqwu.edu.cn

**Keywords:** skeleton editing, ring insertion, ring expansion, heterocycles, ring skeletons

## Abstract

Skeleton editing has rapidly advanced as a synthetic methodology in recent years, significantly streamlining the synthesis process and gaining widespread acceptance in drug synthesis and development. This field encompasses diverse ring reactions, many of which exhibit immense potential in skeleton editing, facilitating the generation of novel ring skeletons. Notably, reactions that involve the cleavage of two distinct rings followed by the reformation of new rings through ring insertion play a pivotal role in the construction of novel ring skeletons. This article aims to compile and systematize this category of reactions, emphasizing the two primary reaction types and offering a thorough exploration of their associated complexities and challenges. Our endeavor is to furnish readers with comprehensive reaction strategies, igniting research interest and injecting fresh impetus into the advancement of this domain.

## 1. Introduction

Organic cyclic compounds are crucial in pharmaceutical science, natural product chemistry, and materials science [1,2]. Cyclic structures significantly impact molecular properties, including lipophilicity, three-dimensional configuration, and rigid scaffolds [3]. Therefore, a fundamental goal in synthetic chemistry is to devise strategies for precisely constructing and manipulating ring systems [4,5]. A prevalent approach among these strategies is executing ring-expansion reactions on existing ring systems [6,7]. This involves inserting one or more atoms into cyclic molecules, enabling skeletal editing. Skeletal editing, a crucial aspect of molecular editing, involves meticulous adjustments to the molecular skeleton, particularly focusing on the ring system [8,9]. Molecular editing reactions are broadly categorized into two types: skeletal editing and peripheral editing [10,11]. Peripheral editing mainly involves activating C–H bonds, while skeletal editing focuses on manipulating ring systems, particularly by adding, deleting, or substituting single or multiple atoms [12,13].

Skeleton editing reactions allow for the rapid construction of new ring structures and modification of core skeletons in molecules, such as aromatic and aliphatic rings, thus achieving post-modification of bioactive compound structures [14,15]. Currently, numerous studies have reported methods for achieving skeleton editing by inserting boron, nitrogen, or carbon atoms, as well as studies on aromatic ring skeleton insertion reactions [16,17]. Skeleton editing, as a direct synthesis method, involves precisely substituting or rearranging atoms within the core ring structure of complex molecules [18,19]. It includes various reaction types, such as single-atom, double-atom, multi-atom, and ring modifications, focusing on altering ring systems [20]. Each modification type involves a wide range of specific chemical reactions, demonstrating remarkable diversity. 

Nevertheless, it is crucial to note that many existing bioactive skeletons cannot undergo this type of skeletal modification. Consequently, it is essential to continually develop more effective and practical techniques. Despite the established nitrogen insertion method, there are still limited methods available for selectively obtaining isomers in skeleton editing [21]. This emphasizes the necessity for further exploration of synthetic strategies in skeletal editing research to expand its applicability and improve modification efficiency [22].

Heterocyclic molecules, serving as the backbone of many bioactive natural products, are prevalent in bioactive compounds [23,24]. The remarkable versatility of these molecules stems from the ability to transform their core ring structure by manipulating two distinct ring structures [25]. However, there is a significant gap in the synthetic approach for constructing novel ring compounds by inserting a smaller ring into the parent ring molecule. This process, referred to as the mutual insertion reaction between rings, is relatively unexplored.

**Figure 1:**
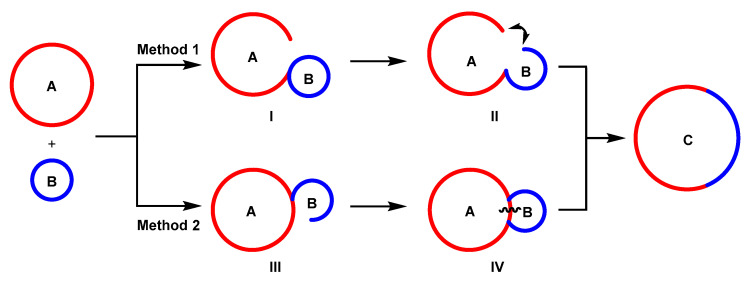
Synthetic strategies of the ring insertion reactions.

After conducting an extensive literature review [20,21,22,23,24,25], we identified two common ring transformation patterns for this reaction type, depicted in Figure 1. Method 1, for rings **A** and **B**, involves disrupting ring **A**, then recombining it with ring **B** at the breakage site, resulting in intermediate **I**. Then, ring **B** dissociates, forming intermediate **II**. Finally, the two disrupted sites are reconnected, forming a new ring **C**. In contrast, method 2 starts by disrupting ring B and combining it with ring **A**, yielding intermediate **III**. Then, ring **B** reconnects with ring **A** at the breakage site, forming intermediate **IV**. Finally, ring **A** dissociates at the connection site, producing a new ring **C**. These reaction types have significant potential for advancing the synthesis and utilization of heterocyclic molecules, expanding their application in various fields.

Such reactions are unique and belong to the category of ring expansion reactions. When consulting the relevant literature, we referred to some review articles [2,7,15,24,25] and further searched for relevant papers based on these publications while fully utilizing our daily accumulation. While our literature collection may not be exhaustive, it still provides insights into the overall characteristics of this reaction type. A detailed analysis of the reaction mechanism reveals that incorporating a ring structure into another heterocyclic ring to form heterocyclic compounds presents intricate challenges. These challenges include managing the activation sequence, precisely controlling chemical selectivity, and navigating regional selectivity [26]. Consequently, the widespread application of this reaction type in synthetic chemistry has been greatly hindered. This reaction requires the cleavage of two distinct rings and the formation of four novel connection sites, thereby increasing the likelihood of isomer formation. Furthermore, as the number of atoms in the ring increases, the distance between these novel connection sites widens, making the reconnection process more challenging. These factors collectively increase the complexity of this reaction and make the overall process highly demanding. However, despite these challenges, the significant advantages of this type of reaction in building new rings are apparent. Directly combining two rings enables efficient and rapid ring formation, thereby achieving a one-step reaction process. This innovative chemical transformation offers a fresh approach to synthesizing heterocyclic compounds containing heteroatoms, providing new impetus for applying these synthetic methods to the creation of pharmacologically active molecules [27,28]. 

## 2. The Formation of a New Ring through Ring Opening and Reconnection

Ring insertion reactions primarily falls within method 1. Typically, this reaction occurs under metal catalysis, wherein one ring undergoes cleavage to generate a metal complex. This complex then engages with the second ring, inducing its rupture, after which the newly formed cleavage site reattaches to produce a novel macrocyclic molecule. To elucidate this concept, specific examples are provided [29]. In these instances, atoms of the original ring are distinguished by various colors, while sites of new bond formation are highlighted with bold bonds.

### 2.1. [5 + 4] Decarboxylative Ring Reconstruction Reaction

**Scheme 1:**
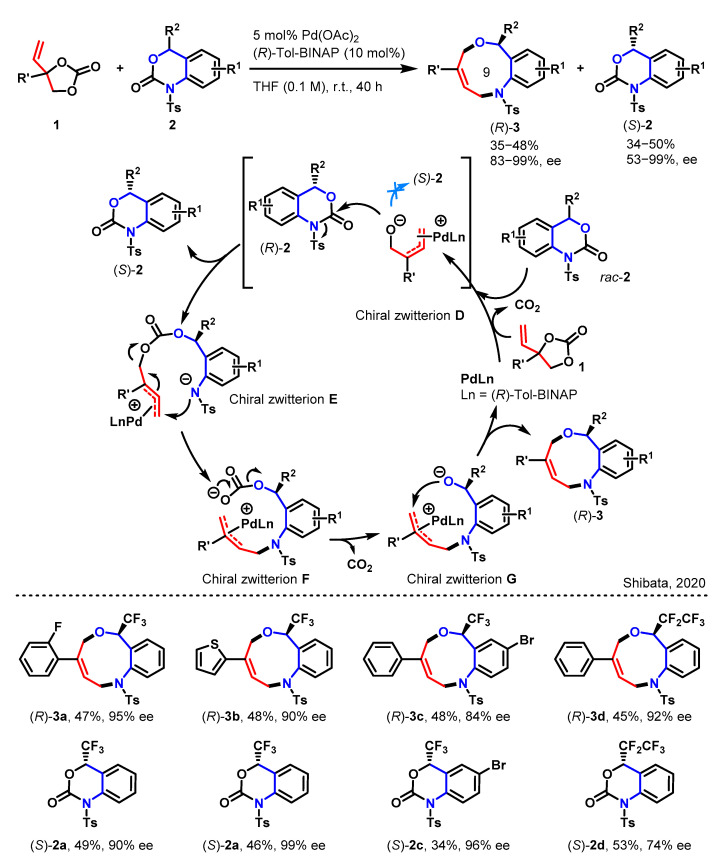
The construction of 9-membered heterocycles via a [5 + 4] annulation [30].

Recently, the Shibata group announced the successful selective synthesis of two enantiomers of trifluoromethyl-benzo[*c*]-[1,5]oxazonines, exhibiting remarkable enantiopurity (Scheme 1) [30,31]. This feat was accomplished via a Pd-catalyzed ring expansion reaction, in which trifluoromethyl-benzo[*d*][1,3]oxazinones reacted with vinyl ethylene carbonates, facilitated by a chiral ligand. As outlined in Scheme 1, the reaction proceeds with remarkable yield and enantioselectivity, yielding (*R*)-**3** products while maintaining the original selectivity of the starting materials (*S*)-**2**. 

The postulated reaction mechanism involves a decarboxylative oxidative addition of Pd^0^Ln (Ln = (*R*)-Tol-BINAP) to compound **1**, resulting in the formation of the chiral Pd-π-allyl zwitterion D. Subsequently, the highly nucleophilic alkoxide oxygen of **D** attacks the carbonyl carbon of (*R*)-**2**, followed by a ring-opening reaction through N–C bond cleavage, generating the chiral zwitterion **E**. Within **E**, the nitrogen atom intramolecularly attacks the terminal sp^2^-hybridized carbon atom of the π-allyl-Pd moiety, leading to the formation of the chiral zwitterion **G** via decarboxylation of **F**. Finally, an intramolecular nucleophilic attack of the oxygen atom on the terminal position of the π-allyl-Pd moiety affords the nine-membered oxazonine (*R*)-**3**, regenerating the Pd/Tol-BINAP complex.

An investigation into the substrate scope was conducted using various substituted compound **2’s**, varying in their substitution patterns on the phenyl ring. The desired CF_3_-benzo[*c*][1,5]oxazonines (*R*)-**3a**–**3d** were obtained in good yields with excellent enantioselectivities, while **2** was recovered with high enantiopurity (up to 99% ee). This study demonstrates an impressive example of realizing the ring insertion reaction between two distinct rings, achieving ring expansion and chirality control.

### 2.2. [6 + 6] Decarboxylative Ring Reconstruction Reaction

**Scheme 2:**
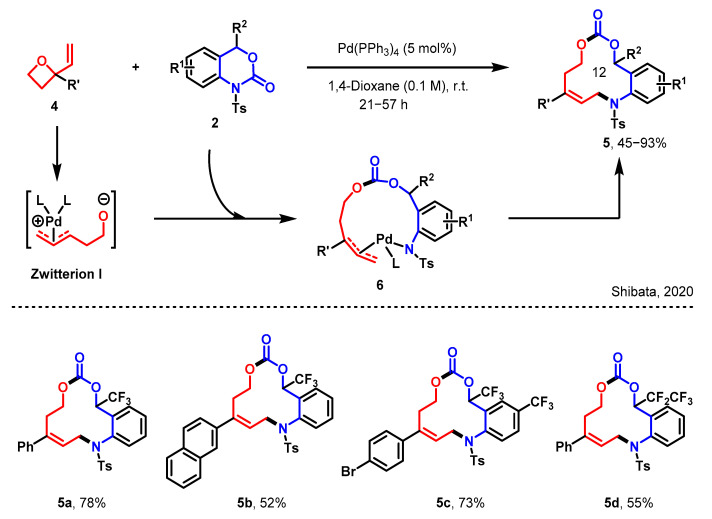
The construction of 12-membered heterocycles via a [6 + 6] annulation [32].

The Shibata group also reported the construction of 12-membered heterocycles through a ring insertion reaction (in Scheme 2) [32]. A novel and efficient method for the synthesis of stereogenic trifluoromethylated 12-membered heterocycles through an unexpected nondecarboxylative Pd-catalyzed ring expansion process of benzo[*d*][1,3]-oxazinones **2** and vinyl oxetanes **4**. These large 12-membered heterocycles contain several functional groups, including a stereogenic CF_3_ carbon center, an amino group, an alkenyl function, and a carbonate moiety, which affords a unique chemical space suitable for drug libraries.

The formation of the Pd complex zwitterion **I**, as initially proposed, was validated by LC–MS spectrometry. This involved the cleavage of the C–N bond in **2**, triggered by a nucleophilic assault from the zwitterion **I** onto the carbonyl moiety. This reaction sequence led to the emergence of the Pd-complex **6**. Subsequently, a reductive elimination occurred between the nitrogen atom and the terminal carbon atom of the Pd-complex **6**, resulting in the formation of the [6 + 6] annulation product **5**. The tolerance of various substituents with varying electronic properties on the benzene ring allowed for the production of corresponding products **5a**–**5c**, yielding them in moderate to good quantities. Notably, even the C_2_F_5_-substituted benzoxazinanone was capable of generating the C_2_F_5_-substituted 12-membered heterocycle **5d**, albeit in a moderate yield.

### 2.3. Nondecarboxylative [6 + 3] and Decarboxylative [4 + 3] Ring Reconstruction Reactions

**Scheme 3:**
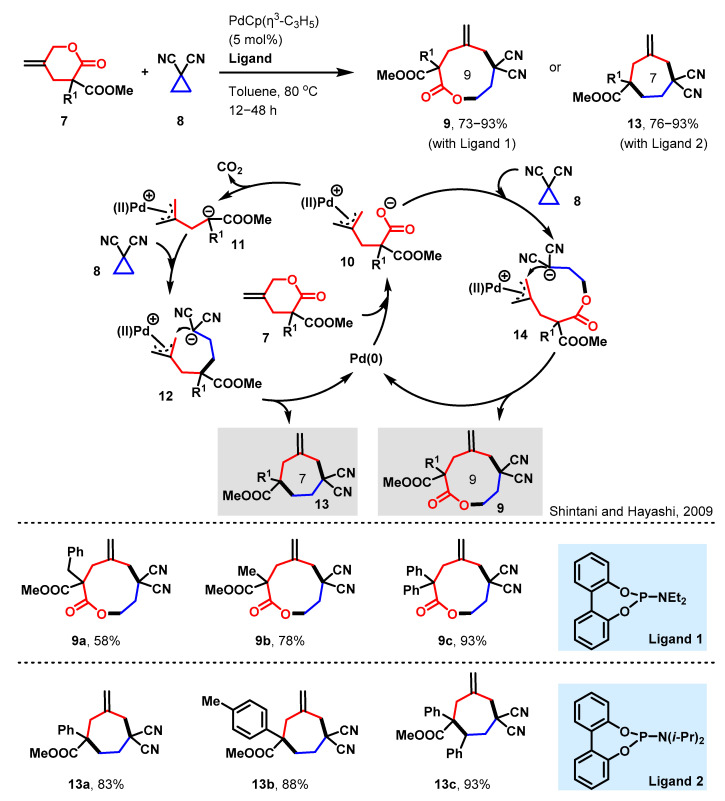
The construction of 9 and 7-membered heterocycles via [6 + 3] and [4 + 3] annulation [33].

Triple rings, being effortlessly operable, are frequently utilized for ring insertion in this particular reaction. As an illustrative instance, Shintani and Hayashi documented a reaction aimed at achieving ring insertion, encompassing both decarboxylation and nondecarboxylation conditions, as detailed in Scheme 3 [33]. This reaction involves a Pd-catalyzed decarboxylative [4 + 3] cyclization of *γ*-methylidene-*δ*-valerolactones with cyclopropanes, converging to produce cycloheptane derivatives. Furthermore, by modifying the electronic properties of the lactone substituents and ligands, the reaction can be seamlessly shifted to nondecarboxylative formal [6 + 3] cyclizations, resulting in the formation of ring-expanded, nine-membered lactones.

The oxidative addition of the allyl ester moiety of **7** to Pd(0) generates π-allylpalladium carboxylate **10**. When R^1^ is an aryl group, it stabilizes the anionic charge on the adjacent carbon through successive decarboxylation, resulting in the formation of the 1,4-zwitterionic species **11**. Subsequently, the anionic carbon of **11** attacks the electrophilic carbon of **8**, yielding intermediate **12**. This intermediate undergoes a ring closure through a nucleophilic attack on the π-allyl palladium moiety, leading to the formation of cycloheptane **13** along with the regeneration of Pd(0). In contrast, when R^1^ is an alkyl group with lesser stabilizing properties, the direct nucleophilic attack of the carboxylate of **10** on **8** occurs more rapidly than decarboxylation, generating intermediate **14**. The ring closure of this intermediate leads to the formation of the 9-membered lactone **9**. Under the specified conditions, the α-alkyl lactones along with lactone afford the ring-expanded nine-membered lactones, specifically **9a**–**c**, with complete selectivity. By employing ligand 2, a diverse set of *γ*-methylidene-*δ*-valerolactones carrying an aryl group at the α-position engage in decarboxylative cyclization reactions with compound **8**, leading to the efficient preparation of compounds **13a**–**13c**.

### 2.4. [3 + n] Cycloadditions of Sulfonyltriazole with Heterocycles 

**Scheme 4:**
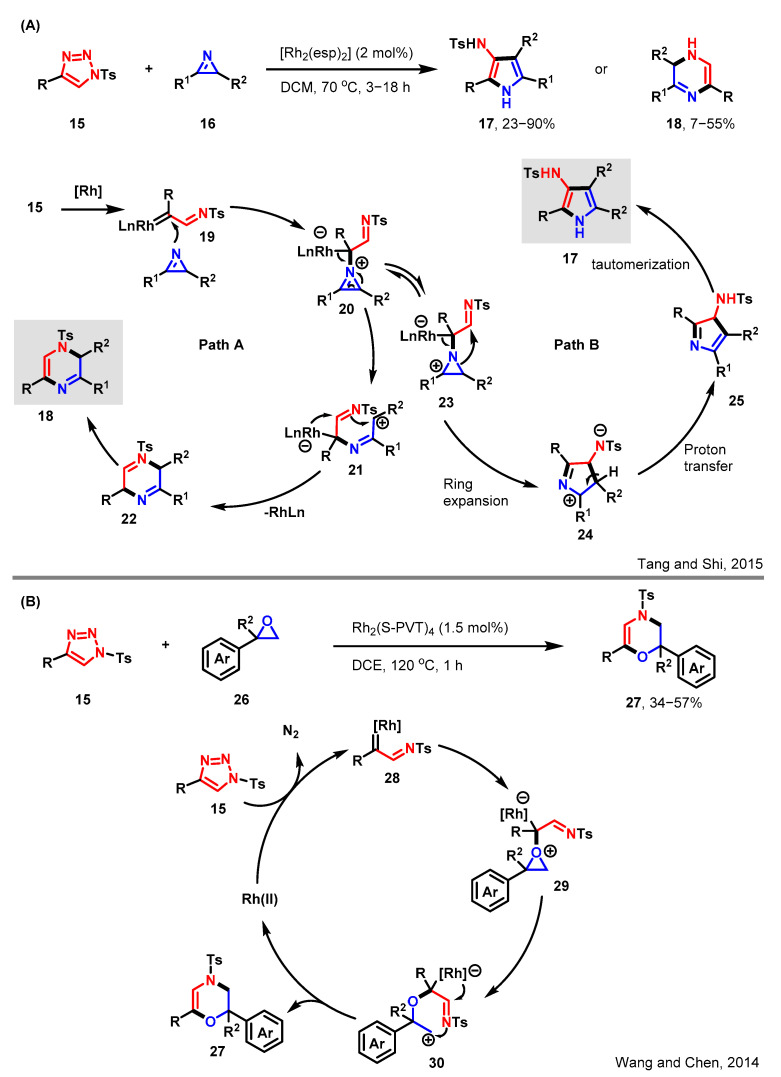
[3 + n] cycloadditions of sulfonyltriazole with ternary ring. (**A**) Reaction of sulfonyltriazoles with azirines [35]; (**B**) Reaction of sulfonyltriazoles with oxetanes [36].

Owing to the chemical similarity between sulfonyl triazole and diazo compounds, sulfonyl triazole undergoes facile dissociation catalyzed by Rh(II), resulting in the formation of a metal complex [34]. Subsequently, this complex engages in ring-insertion reactions with the cleaved products of C–S, C–C, C–N, and C–O bonds present in another ring, leading to the generation of ring-insertion products. In subsequent sections, we will delve into this matter in greater detail. Initially, our focus will be on two illustrative examples of ternary ring cleavage and insertion reactions, as schematically represented in Scheme 4.

Sulfonyl-1,2,3-triazoles, being precursors of Rh-azavinyl carbenes, have attracted significant research attention recently [37]. Azavinyl carbenes are highly desirable intermediates in the synthesis of cyclic derivatives, particularly *N*-heterocycles, through [3 + n] cycloadditions [38,39]. Tang and Shi described the Rh(II)-catalyzed intermolecular [3 + 2] and [3 + 3] cycloaddition reactions of 2*H*-azirine **16** with *N*-sulfonyl-1,2,3-triazole **15**, resulting in the synthesis of functionalized pyrrole **17** and 2*H*-pyrazine **18**, as outlined in Scheme 4A [35]. In the presence of the Rh(II) catalyst, *N*-sulfonyltriazole **15** generates an azavinyl carbene intermediate **19**. This Fisher-type carbene **19** readily accepts nucleophilic attack from the nitrogen atom of 2*H*-azirine, resulting in the formation of the key zwitterionic intermediate **20**. Depending on the reaction path, either pyrrole or pyrazine derivatives can be obtained. Path A involves a ring-opening process of intermediate **21**, followed by a 6π electrocyclization to yield the final pyrazine derivative **18**. During the synthesis of pyrrole, intermediate **20** exists in a state of equilibrium with its tautomer, **23**. Following path B, a subsequent ring expansion of **23** leads to the formation of intermediate **24**. Subsequently, the aromatization of intermediate **25** culminates in the formation of the desired final product, pyrrole **17**. The reactivity of intermediate **20** is significantly influenced by the properties of R^1^ and R^2^. In most cases, path A dominates the reaction. However, when R^1^ is electronegative or R^2^ is a sterically bulky group, the azirine ring becomes more unstable or strained, enabling the ring-opening process and thus leading to the formation of both **17** and **18** in these reactions.

Small heterocyclic compounds, including ternary and quaternary heterocyclics, carry high ring strain, predisposing them to undergo ring-opening and ring-expansion reactions [40]. The reaction mechanism behind this involves the interaction of diazo compounds with metals like copper (Cu) and rhodium (Rh), resulting in the formation of metal carbenes [41,42]. These metal carbenes, being electrophilic, can engage in reactions with oxygen atoms found in heterocycles, leading to the formation of carbonyl ylides. Subsequently, these intermediates can participate in reactions such as the Stevens rearrangement, ultimately yielding ring-expanded products. Following a similar reaction mechanism, Wang and Chen described a Rh(II)-catalyzed ring-expansion reaction between sulfonyl triazoles and oxetanes [36]. This catalytic process, through the ring insertion reaction, enables the synthesis of a range of ring-expanded oxetane derivatives, as elegantly depicted in Scheme 4B. The reaction involves the utilization of a well-orchestrated reaction between epoxides and triazoles. The reaction of triazole **15** with dirhodium tetracarboxylate gives rise to *α*-imino rhodium(II) carbene species **28**, which is catalyzed by the rhodium and accompanied by the expulsion of a dinitrogen molecule. Subsequently, a nucleophilic attack by the epoxide oxygen on the electrophilic carbenoid carbon center leads to the formation of oxonium species **29**. This process is driven by the combined effects of electrophilic activation and the ring strain within the epoxide. Following this, a C–O cleavage occurs, generating a carbocationic intermediate **30**. Finally, a nucleophilic attack by the imino group on the carbocation results in the formation of the desired final product **27**, along with the liberation of the dirhodium complex. This method represents a novel and effective approach for the preparation of these substituted 3,4-dihydro-2*H*-1,4-oxazine derivatives. 

**Scheme 5:**
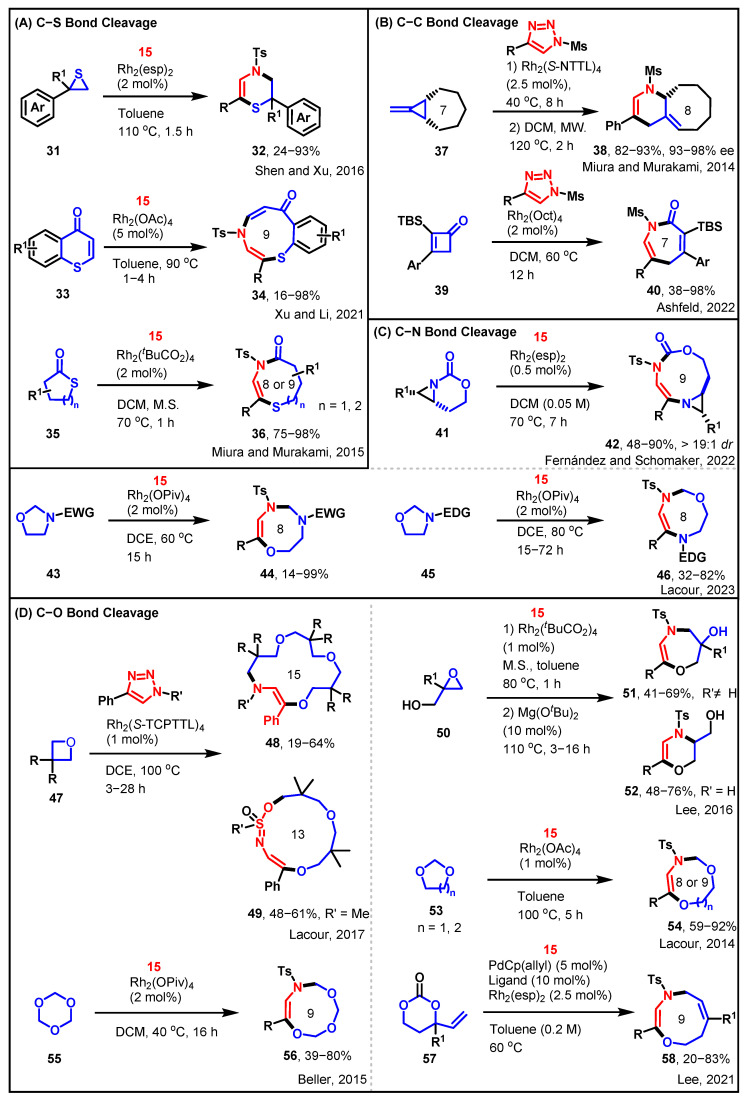
[3 + n] cycloadditions of sulfonyltriazole with heterocyclics [43,44,45,46,47,48,49,50,51,52,53,54].

Upon the intensification of studies regarding this reaction type, the original range of small heterocyclic compounds has undergone a rapid transition towards a more diverse array of larger heterocyclic compounds. Catalysts, predominantly Rh or Pd, initially transform *N*-sulfonyl-1,2,3-triazole into electrophilic iminocarbene, subsequently merging with heterocyclic compounds to generate intermediates. This process culminates in the ring expansion reaction through the cleavage of heterocyclic rings and the formation of novel rings. These reactions share a similar mechanistic framework, with exemplary reactions compiled and presented in Scheme 5. Analysis of various heterocyclic ring cleavages reveals patterns in C–S, C–C, C–N, and C–O bond cleavages. Notably, the scalability of this reaction type extends to larger heterocyclic systems. Furthermore, the involvement of heterocyclic rings is not restricted to a single ring, as reactions may involve two or more heterocyclic rings. 

Sulfur heterocycles and oxiane exhibit structural similarities and can engage in ring insertion reactions akin to those exhibited by oxiane. Shen and Xu successfully demonstrated the Rh-catalyzed regioselective coupling of thiirane **31** with *N*-sulfonyl-1,2,3-triazole **15**, resulting in the formal [3 + 3] cycloaddition product, 3,4-dihydro-2*H*-1,4-thiazine **32** [43]. This reaction proceeded via the 1,3-insertion mechanism of azavinyl carbene, as outlined in Scheme 5A. Xu and Li proposed another sample of a synthetic protocol for the preparation of the 9-membered N,S-heterocycle thiazonine through the cleavage of the C–S bond [44]. A straightforward synthesis of the multi-functionalized benzothiazonine **34** was achieved by employing an Rh-catalyzed denitrogenative annulation reaction between *N*-sulfonyl-1,2,3-triazole **15** and thiochromone **33**. By considering cyclic thioester (thiolactone) **35** as the substrate, an analogous acyl migration process could potentially expand the ring size by three atoms, leading to the formation of a medium-sized lactam **36** containing a vinyl sulfanyl moiety [45].

In the ring insertion reaction involving the cleavage of the C–C bond within the ring, the current focus is on smaller rings that exhibit greater ring strain in Scheme 5B. Miura and Murakami introduced a method that entails the initial enantioselective installation of a cyclopropane ring onto methylenecyclopropanes, followed by a thermal skeletal rearrangement process [46]. This process involves the opening of both the newly installed and the preexisting cyclopropane rings. Specifically, cyclopropane **37** underwent ring opening to afford compound **38**. The Ashfeld group reported an alternative approach to C–C bond cleavage within the ring [47]. They developed an intermolecular rhodium(II) catalyzed formal [4 + 3] cycloaddition reaction between vinyl ketenes **39** and triazoles. This method offers a synthetic route to azepinones **40** that possesses a range of translational possibilities. 

The employment of *N*-sulfonyl-1,2,3-triazoles as carbene precursors effectively facilitated the enlargement of the bicyclic aziridine ring (**41**) into densely substituted dehydropiperazine, ultimately yielding the expanded [3,9]-bicyclic aziridine **42** (Scheme 5C) [48]. Rigorous computational analysis indicated that the presence of a significant rotational barrier around the C–N bond in the lower-energy conformer of the aziridinium ylide intermediate enabled an alternative synthetic pathway. This approach offered efficient access to [3,9]-bicyclic aziridines with impressive yields and diastereoselectivity. Additionally, the Lacour group reported that the treatment of 5-membered oxazolidine precursors with *N*-sulfonyl-1,2,3-triazoles, catalyzed by dirhodium, provided a regiodivergent approach for the synthesis of either 8-membered 1,3,6- or 1,4,6-oxadiazocines (**44** and **46**), depending on the substitution pattern of the nitrogen atom (Scheme 5C) [49]. Both examples involved the cleavage of C–N bonds within the ring, followed by a [3 + n] cycloaddition reaction with *N*-sulfonyl-1,2,3-triazole **15**. 

The reaction can produce both small and large rings, and more interestingly, it can also continuously achieve cyclization reactions of two or three rings, further expanding the boundaries of synthetic chemistry (Scheme 5D). Lacour reported that 15- and 13-membered ring aza-macrocycles (**48** and **49**) can be selectively prepared via formal [3 + 4 + 4 + 4] and [5 + 4 + 4] condensations of *ɑ*-imino carbenes and oxetanes under Rh(II)-catalysis or thermal activation, using *N*-sulfonyl triazoles and oxetanes as substrates [50]. The sequential addition of two and three oxetanes is primarily due to the requirement for S_N_2-like transition states and/or trans-annular strain. Lee and his team reported a novel Rh(II)/Mg(O*^t^*Bu)_2_-catalyzed tandem one-pot reaction for the synthesis of 1,4-oxazepine (**51**) and 1,4-oxazine (**52**) derivatives from *N*-sulfonyl-1,2,3-triazoles and glycidols, involving sequential O–H insertion of rhodium(II) carbene and subsequent Lewis acid-catalyzed regioselective epoxide ring-opening reaction [51]. The Lacour group reported that 8- and 9-membered dioxazocines (**54**) can be readily synthesized from *N*-sulfonyl-1,2,3-triazoles in a single-step procedure under dirhodium catalysis, using 1,3-dioxolane and 1,3-dioxane as solvents and reagents [52]. The Beller group reported that the rhodium(II)-catalyzed denitrogenative coupling of *N*-sulfonyl-1,2,3-triazoles with 1,3,5-trioxane leads to 9-membered ring trioxazonines (**56**) in moderate to good yields [53]. The key step in the reaction involves 1,3,5-trioxane acting as an oxygen nucleophile, reacting with the α-aza-vinylcarbene intermediate, resulting in ylide formation. The Lee group reported a synergistic palladium(0)/rhodium(II) dual catalytic cycloaddition of vinylpropylene carbonates with *N*-sulfonyl-1,2,3-triazoles in a formal [6 + 3] dipolar cycloaddition to produce 9-membered oxazonines (**58**) [54]. 

After thorough deliberation, we discovered that the insertion reaction between rings typically adheres to a precise mechanism within a metal-catalyzed environment. Initially, one ring opens, and one of its ends attaches to another ring. Subsequently, the second ring opens and merges with the first, ultimately forming a larger ring. Within this sequence of reactions, the most crucial and demanding step is the final ring-closing reaction. Notably, as the number of atoms within the reacting rings increases, the complexity of ring closure also escalates. Despite these challenges, this type of ring insertion reaction has garnered significant research interest owing to the utilization of leaving groups or easily cleavable moieties.

## 3. The Formation of a New Ring through Ring Closure and then Ring Opening

Next, we turn our attention to another ring insertion reaction with method 2, where ring expansion primarily occurs through the parallel connection of two small rings. Subsequently, the bond at the connection point undergoes cleavage, resulting in the formation of a larger ring. A cursory review of the available literature reveals that reports on this reaction type are comparatively scarce in comparison to the previously discussed ones.

### 3.1. [3 + 3] and [3 + 4] towards Diverse Azaheterocycles

**Scheme 6:**
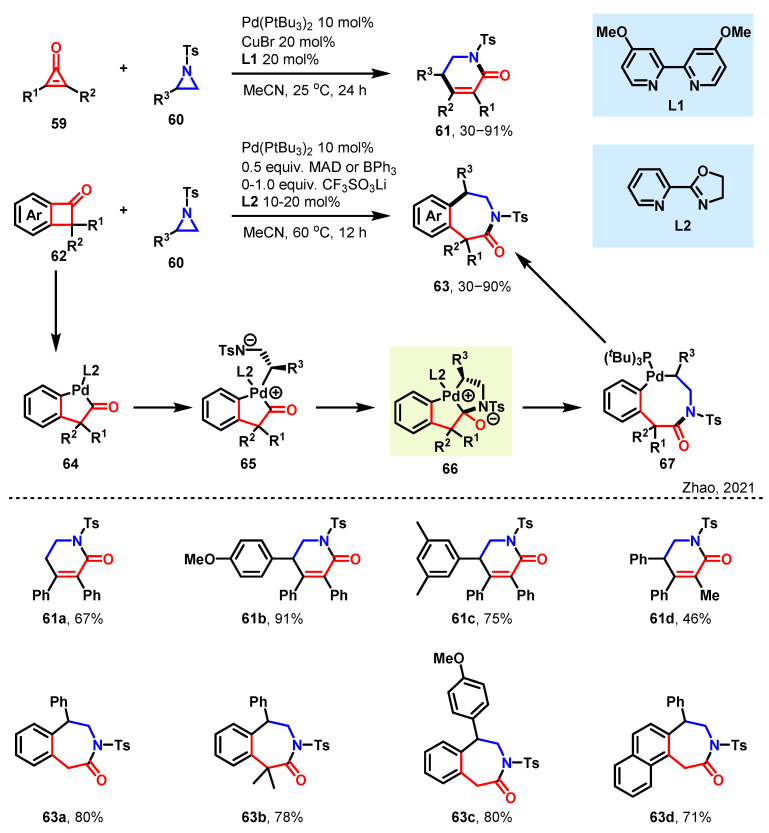
[3 + 3] and [3 + 4] towards diverse 6- and 7-membered azaheterocycles [55].

Zhao and coworkers have described a synthetically useful and mechanistically intriguing ring expansion strategy in which two different strained rings can be cross-dimerized by intertwining the strain-release-driven oxidative C–C bond cleavage and C–N bond cleavage, which opens up a substantial chemical space for *N*-heterocycle synthesis in a stereospecific manner (in Scheme 6) [55]. The reaction can be initiated by the oxidative C–C bond cleavage of benzocyclobutenone **62** to give a 5-membered palladacycle **64**, which would undergo a further oxidative addition of aziridine **60** by an S_N_2 mechanism, subsequent nucleophilic addition of the C=O group, and reductive acylation to give intermediate **65**. The anionic amido group on **65** would attack the carbon atom of the carbonyl group to generate the zwitterion complex **66** with C–N bond formation. The structure of this intermediate can be considered the result of the union of two rings. Further undergoes the reductive acylation to give intermediate **67**. Followed by reductive elimination to deliver the final product **63** and regenerate the Pd(0) catalyst. The role of the Lewis acid is proposed to be twofold: first, it could facilitate C–C or C–N bond cleavage by coordinating with the carbonyl or sulfonate group on substrate **60** or **62**. Second, the Lewis acid could also accelerate the nucleophilic addition of the anionic amido group on aza-organometal species **65** to the carbonyl groups. 

Under optimized conditions, they were pleased to observe that the reaction proceeded with 99% enantiospecificity. This finding indicated that the mechanism underlying the reaction between aziridines **60** and cyclopropenone **59** was analogous to that observed in the reaction between aziridines **60** and benzocyclobutenones **62**. When examining the scope of the aziridine coupling partner under these modified conditions, it was determined that a range of dihydropyridinones (**61a**–**61d**) could be obtained in satisfactory yields (Scheme 6). Notably, benzocyclobutenones bearing substituents at any position on the benzene ring effectively yielded the corresponding 7-membered benzoazepinones (**63a**–**63d**). This methodology facilitates the modular and robust synthesis of 3-benzazepinones, dihydropyridinones, and uracils, which are versatile building blocks in numerous pharmaceutical and biologically active compounds. During the reciprocal insertion of two rings, the quantity of atoms involved in the reduction remains unchanged. Furthermore, the overall count of atoms within the enlarged ring that has been recently formed is identical to the combined number of atoms from the two smaller rings initially present, thereby emphasizing the benefits associated with this particular reaction.

### 3.2. Expansion of Cyclic Ketones to Macrolactones

Zuo and his team have made a remarkable breakthrough in the field of cyclic ketones by developing a rapid and straightforward ring expansion protocol [56]. This protocol, which employs visible light irradiation and aerobic conditions, effectively utilizes the widely accessible cerium triflate in conjunction with a cyanoanthracene co-catalyst. This innovative method has enabled the preparation of a diverse collection of over 100 macrolactones, with ring systems ranging from 9- to 19-membered macrocycles, derived from basic building blocks. The protocol’s simplicity and efficiency are noteworthy achievements in the field [41,57].

**Scheme 7:**
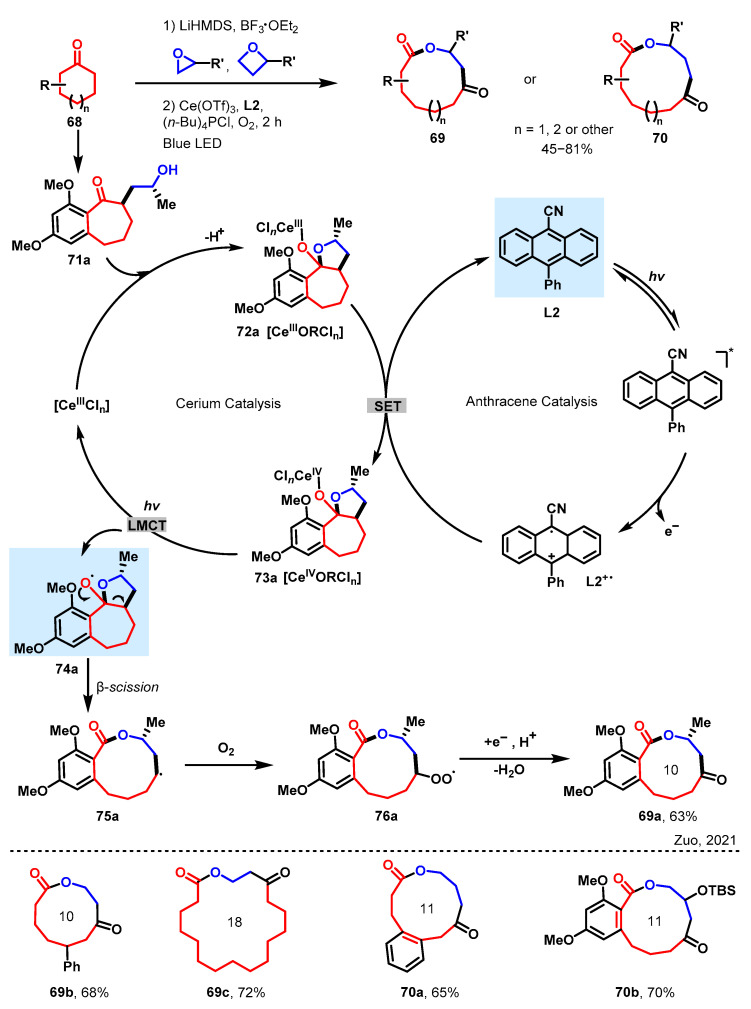
Ring expansion protocol of cyclic ketones to macrolactones [56]. * The excited-state of compound **L2**.

Scheme 7 outlines a plausible dual catalytic cycle. The preparation of compound **71a** involves a straightforward enolate alkylation of substituted cycloheptanone with ethylene oxide. The Lewis acidic nature of Ce^III^ enhances the formation of lactol **72a** in the ketone/lactol equilibrium. Subsequently, the in situ-formed Ce^III^ lactol complex is smoothly oxidized by L2^+•^ to its Ce^IV^ state, generating intermediate **73a**. This intermediate is photoexcited to produce the O-centered radical **74a**, which facilitates ring expansion. The resulting alkyl radical **75a** is promptly trapped by oxygen, yielding a peroxyl radical **76a**. The peroxyl radical can be further quenched by photoexcited cyanoanthracene, regenerating L2^+•^, and the desired keto-macrolactone **69a**. Given the rapid kinetics of LMCT-homolysis, the resting state of the cerium cycle would be Ce^III^ complexes. The introduction of a photoinduced electron transfer catalyst further accelerates its oxidation, highlighting the innovative and efficient nature of this protocol. The cyclic ketones, epoxides, and oxetanes employed in these syntheses are easily accessible from commercial sources. In terms of practical applications, the integration of cyclohexanones and epoxides provides a comprehensive and efficient method for synthesizing macrolactones with up to 19 members, such as **69a**–**70b** (as shown in Scheme 7). This approach effectively demonstrates the construction of macrolactones with polycyclic scaffolds.

### 3.3. Expansion of Thiophenes by Bicyclobutane Insertion

**Scheme 8:**
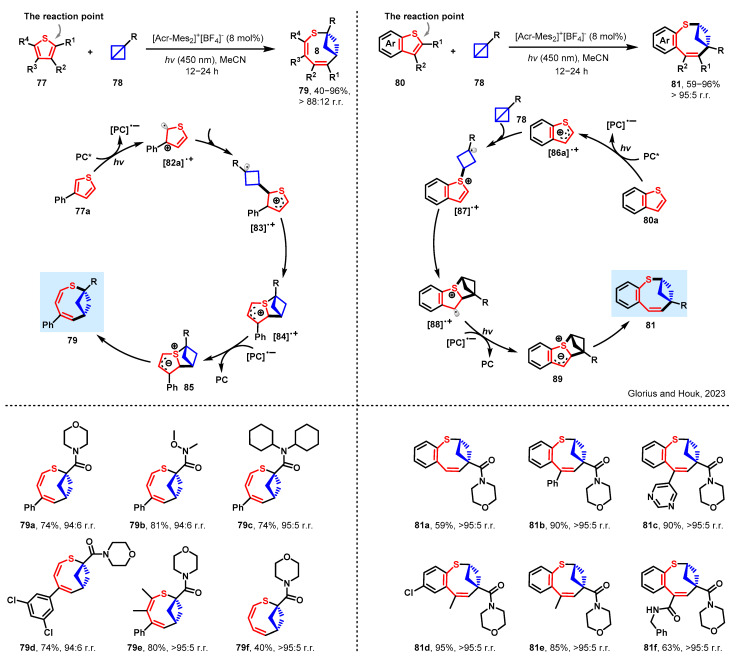
Ring expansion of thiophenes by bicyclobutane insertion [58]. PC* is the excited-state of photocatalyst.

The thiophene molecular structure, a significant class of compounds, is reflected in numerous drug molecules. In their quest to develop an innovative method for synthesizing bicyclic compounds, the researchers utilized the ring insertion protocol, which entails the insertion of a smaller ring into the thiophene molecule. After extensive research and practice, Glorius and Houk achieved a remarkable breakthrough in this synthetic strategy. They reported a photoinduced dearomative ring enlargement of thiophenes by inserting bicyclo[1.1.0]butanes to produce eight-membered bicyclic rings under mild conditions in Scheme 8 [58]. The synthetic value, broad functional-group compatibility, and excellent chemo- and regioselectivity were demonstrated through scope evaluation and product derivatization.

Experimental studies have demonstrated that PC* reacts with **77a**, leading to the formation of the radical cation intermediate designated as [**82a**]^•+^. The bicyclo[1.1.0]butane insertion process for [**82a**]^•+^ involves a transition state that exhibits a free energy barrier of 12.6 kcal/mol. This results in the formation of the thermodynamically more stable species [**83**]^•+^. Subsequently, the kinetic and thermodynamic favorability of the C–S bond formation leads to the production of the heterocycle intermediate [**84**]^•+^. The photocatalyst-mediated reduction of [**85**]^•+^ is favored thermodynamically by 11.5 kcal/mol. The ultimate C–S bond cleavage proceeds with kinetic ease, leading to the irreversible formation of product **79**. Alternatively, during benzothiophene expansion, which occurs with inverted regioselectivity to produce **81**, the spin density distribution of the radical cation intermediate [**86a**]^•+^ differs from that of [**82a**]^•+^. Notably, the reaction mechanism involving benzothiophene **80a** (Scheme 8) differs from that with 3-phenylthiophene **77a**. The bicyclo[1.1.0]butane insertion (**84**) into the S-radical exhibits a similar free energy barrier to **83**. Furthermore, the subsequent C–C bond formation for **88** represents the rate-limiting transition state. The formation of the fused heterocycle **89** radical cation is exergonic. The cleavage of the C–S bond to obtain product **81** is believed to proceed with kinetic ease.

Scheme 8 exhibits the compatibility of diverse cyclic substituted bicyclo[1.1.0]butanes with the transformation process, leading to the production of corresponding products in high yields and regioselectivities (**79a**–**79f**). Following this, an investigation into the substrate scope of benzothiophene derivatives was undertaken, revealing contrasting regioselectivities in comparison to those noted for thiophenes (**81a**–**81f**). The synthetic worth, coupled with the extensive compatibility of functional groups and remarkable chemo- and regioselectivity, was confirmed through a scope assessment and product derivatization analysis.

## 4. Conclusions and Perspectives

Through the collection and analysis of the cases of ring insertion reactions, we have summarized two main types of insertion reactions. One is that after the two rings are connected, the two ends of the two rings are broken and reconnected to form a new ring. The other is that after the two rings are connected, the connection bond is broken to form a new ring. Notably, examples of these two types of ring insertion reactions are infrequent, indicating their significant challenges. The selection of raw materials is limited, and the reaction process is fraught with synthetic difficulties, such as activation order, chemical selectivity, and regional selectivity. These complexities hinder the advancement of ring insertion heterocyclic compound reactions.

Despite these limitations, ring insertion reactions exhibit remarkable synthetic potential. For instance, the utilization of triazole compounds enables the insertion of multiple ring types, enabling the synthesis of rings ranging from small to large. Currently, there is a concerted effort to explore more diverse and intricate ring systems, indicating ample research opportunities for various triazole ring compounds.

It is noteworthy that bicyclo[1.1.0]butanes have been successfully inserted into thiophenes, achieving the one-step synthesis of bridged molecules through ingenious design. This accomplishment offers additional possibilities for ring insertion reactions. Given this progress, we are optimistic that in the future, more bridge compounds resembling bicyclo[1.1.0]butanes will react with diverse bridge compounds, yielding an expanded array of complex natural product analogues. Through this discussion, we aim to spark the interest of researchers, kindle enthusiasm for this field, and jointly promote the research progress of ring insertion reactions.

## References

[1] Yang N.-N., Ma Q.-Y., Yang L., Xie Q.-Y., Dai H.-F., Yu Z.-F., Zhao Y.-X. (2024). A New Macrolide from the Strain *Cordyceps* spp. from Cell Fusion between Cordyceps militaris and Cordyceps cicadae. Chem. Nat. Compd..

[2] Escolano M., Gaviña D., Alzuet-Piña G., Díaz-Oltra S., Sánchez-Roselló M., del Pozo C. (2024). Recent Strategies in the Nucleophilic Dearomatization of Pyridines, Quinolines, and Isoquinolines. Chem. Rev..

[3] Buttard F., Vigier J., Lebel H., Besset T. (2024). C-Centered radical intermediates for C(sp^3^)-H bonds functionalization: An emerging approach towards alkyl thiocyanates. Eur. J. Org. Chem..

[4] Li J., Amatuni A., Renata H. (2020). Recent advances in the chemoenzymatic synthesis of bioactive natural products. Curr. Opin. Chem. Biol..

[5] Zhu Q., Liu C. (2021). The future directions of synthetic chemistry. Pure Appl. Chem..

[6] Yang Z., Zalessky I., Epton R.G., Whitwood A.C., Lynam J.M., Unsworth W.P. (2023). Ring Expansion Strategies for the Synthesis of Medium Sized Ring and Macrocyclic Sulfonamides. Angew. Chem. Int. Ed..

[7] Donald J.R., Unsworth W.P. (2017). Ring-Expansion Reactions in the Synthesis of Macrocycles and Medium-Sized Rings. Chem. Eur. J..

[8] Cheng Q., Bhattacharya D., Haring M., Cao H., Mück-Lichtenfeld C., Studer A. (2024). Skeletal editing of pyridines through atom-pair swap from CN to CC. Nat. Chem..

[9] Jurczyk J., Woo J., Kim S.F., Dherange B.D., Sarpong R., Levin M.D. (2022). Single-atom logic for heterocycle editing. Nat. Synth..

[10] Liu S., Yang Y., Song Q., Liu Z., Lu Y., Wang Z., Sivaguru P., Bi X. (2024). Tunable molecular editing of indoles with fluoroalkyl carbenes. Nat. Chem..

[11] Kennedy S.H., Dherange B.D., Berger K.J., Levin M.D. (2021). Skeletal editing through direct nitrogen deletion of secondary amines. Nature.

[12] Liu Z., Sivaguru P., Ning Y., Wu Y., Bi X. (2023). Skeletal Editing of (Hetero)Arenes Using Carbenes. Chem. Eur. J..

[13] Joynson B.W., Ball L.T. (2023). Skeletal Editing: Interconversion of Arenes and Heteroarenes. Helv. Chim. Acta.

[14] Zippel C., Seibert J., Bräse S. (2021). Skeletal Editing-Nitrogen Deletion of Secondary Amines by Anomeric Amide Reagents. Angew. Chem. Int. Ed..

[15] Liu F., Anand L., Szostak M. (2023). Diversification of Indoles and Pyrroles by Molecular Editing: New Frontiers in Heterocycle-to-Heterocycle Transmutation. Chem. Eur. J..

[16] Reisenbauer J.C., Green O., Franchino A., Finkelstein P., Morandi B. (2022). Late-stage diversification of indole skeletons throughnitrogen atom insertion. Science.

[17] Bartholomew G.L., Carpaneto F., Sarpong R. (2022). Skeletal Editing of Pyrimidines to Pyrazoles by Formal Carbon Deletion. J. Am. Chem. Soc..

[18] Hui C., Wang Z., Wang S., Xu C. (2022). Molecular editing in natural product synthesis. Org. Chem. Front..

[19] Bartholomew G.L., Kraus S.L., Karas L.J., Carpaneto F., Bennett R., Sigman M.S., Yeung C.S., Sarpong R. (2024). ^14^N to ^15^N Isotopic Exchange of Nitrogen Heteroaromatics through Skeletal Editing. J. Am. Chem. Soc..

[20] Unsworth W.P., Avestro A.-J. (2021). Unprecedented reactionsfor molecular editing. Nature.

[21] He Y., Wang J., Zhu T., Zheng Z., Wei H. (2024). Nitrogen atom insertion into arenols to access benzazepines. Chem. Sci..

[22] Qiu X., Sang Y., Wu H., Xue X.-S., Yan Z.-Z., Wang Y., Cheng Z., Wang X., Tan H., Song S. (2021). Cleaving arene rings for acyclic alkenylnitrile synthesis. Nature.

[23] Chaudhary A. (2021). Recent development in the synthesis of heterocycles by 2-naphthol-based multicomponent reactions. Mol. Div..

[24] Biletskyi B., Colonna P., Masson K., Parrain J.-L., Commeiras L., Chouraqui G. (2021). Small rings in the bigger picture: Ring expansion of three- and four-membered rings to access larger all-carbon cyclic systems. Chem. Soc. Rev..

[25] Huber T., Wildermuth R.E., Magauer T. (2018). 9-Membered Carbocycles: Strategies and Tactics for their Synthesis. Chem. Eur. J..

[26] Sindlinger M., Ströbele M., Grunenberg J., Bettinger H.F. (2023). Accessing unusual heterocycles: Ring expansion of benzoborirenes by formal cycloaddition reactions. Chem. Sci..

[27] Baidya R., Das P., Pratihar P., Maiti D.K. (2023). Ring expansion and fused cyclization catalysis to construct indoloquinazolinones with functionalization. Chem. Commun..

[28] Babar K., Zahoor A.F., Ahmad S., Akhtar R. (2021). Recent synthetic strategies toward the synthesis of spirocyclic compounds comprising six-membered carbocyclic/heterocyclic ring systems. Mol. Div..

[29] Stephens T.C., Unsworth W.P. (2020). Consecutive Ring-Expansion Reactions for the Iterative Assembly of Medium-Sized Rings and Macrocycles. Synlett.

[30] Uno H., Punna N., Tokunaga E., Shiro M., Shibata N. (2020). Synthesis of Both Enantiomers of Nine-Membered CF_3_-Substituted Heterocycles Using a Single Chiral Ligand: Palladium-Catalyzed Decarboxylative Ring Expansion with Kinetic Resolution. Angew. Chem. Int. Ed..

[31] Das P., Gondo S., Nagender P., Uno H., Tokunaga E., Shibata N. (2018). Access to benzo-fused nine-membered heterocyclic alkenes with a trifluoromethyl carbinol moiety via a double decarboxylative formal ring-expansion process under palladium catalysis. Chem. Sci..

[32] Uno H., Imai T., Harada K., Shibata N. (2020). Synthesis of Highly Functionalized 12-Membered Trifluoromethyl Heterocycles via a Nondecarboxylative Pd-Catalyzed [6 + 6] Annulation. ACS Catal..

[33] Shintani R., Murakami M., Tsuji T., Tanno H., Hayashi T. (2009). Palladium-Catalyzed Decarboxylative [4 + 3] Cyclization of *γ*-Methylidene-*δ*-valerolactones with 1,1-Dicyanocyclopropanes. Org. Lett..

[34] Yuan W., Xu J. (2021). Ring Expansions of Oxetanes. Chin. J. Org. Chem..

[35] Zhao Y.-Z., Yang H.-B., Tang X.-Y., Shi M. (2015). Rh^II^-Catalyzed [3 + 2] Cycloaddition of 2*H*-Azirines with *N*-Sulfonyl1,2,3-Triazoles. Chem. Eur. J..

[36] Ma X., Pan S., Wang H., Chen W. (2014). Rhodium-Catalyzed Transannulation of *N*-Sulfonyl-1,2,3-triazoles and Epoxides: Regioselective Synthesis of Substituted 3,4-Dihydro-2*H*-1,4-oxazines. Org. Lett..

[37] Mishra D.R., Chakroborty S., Lalitha P., Panda B.S., Mishra N.P., Barik A., Panda A.R., Malviya J., Khatarkar K., Mukopadhyay M. (2024). Advances in Metal-Catalyzed Denitrogenative Pathways for N-Heterocycle Synthesis. Top Catal..

[38] Wang Y., Lei X., Tang Y. (2015). Rh(II)-catalyzed cycloadditions of 1-tosyl 1,2,3-triazoles with 2*H*-azirines: Switchable reactivity of Rh-azavinylcarbene as [2C]- or aza-[3C]-synthon. Chem. Commun..

[39] Ding H., Hong S., Zhang N. (2015). Rhodium(II)-catalyzed transannulation of 1-sulfonyl-1,2,3-triazoles with 2*H*-azirines: A new method to dihydropyrazines. Tetrahedron Lett..

[40] Liu Z.-K., Gao Y., Hu X.-Q. (2021). Recent advances in catalytic synthesis of mediumring lactones and their derivatives. Catal. Sci. Technol..

[41] Zhao X., Zhang Y., Wang J. (2012). Recent developments in copper-catalyzed reactions of diazo compounds. Chem. Commun..

[42] Yang D., Guan Z., Peng Y., Zhu S., Wang P., Huang Z., Alhumade H., Gu D., Yi H., Lei A. (2023). Electrochemical oxidative difunctionalization of diazo compounds with two different nucleophiles. Nat. Commun..

[43] Lu X.-L., Liu Y.-T., Wang Q.-X., Shen M.-H., Xu H.-D. (2016). Straightforward regioselective construction of 3,4-dihydro-2*H*-1,4-thiazine by rhodium catalysed [3 + 3] cycloaddition of thiirane with 1-sulfonyl-1,2,3-triazole: A pronounced acid additive effect. Org. Chem. Front..

[44] Jablasone S.T., Ye Z., Duan S., Xu Z.-F., Li C.-Y. (2021). Synthesis of benzothiazonine by rhodiumcatalyzed denitrogenative transannulation of 1-sulfonyl-1,2,3-triazole and thiochromone. Org. Biomol. Chem..

[45] Miura T., Fujimoto Y., Funakoshi Y., Murakami M. (2015). A Reaction of Triazoles with Thioesters to Produce β-Sulfanyl Enamides by Insertion of an Enamine Moiety into the Sulfur-Carbonyl Bond. Angew. Chem. Int. Ed..

[46] Miura T., Nakamuro T., Liang C.-J., Murakami M. (2014). Synthesis of trans-Cycloalkenes via Enantioselective Cyclopropanation and Skeletal Rearrangement. J. Am. Chem. Soc..

[47] Hill H.M., Tucker Z.D., Rodriguez K.X., Wendt K.A., Ashfeld B.L. (2022). Generation of Functionalized Azepinone Derivatives via a (4 + 3)-Cycloaddition of Vinyl Ketenes and α-Imino Carbenes Derived from *N*-Sulfonyl-triazoles. J. Org. Chem..

[48] Dequina H.J., Eshon J., Schmid S.C., Raskopf W.T., Sanders K.M., Fernández I., Schomaker J.M. (2022). Re-Evaluation of Product Outcomes in the Rh-Catalyzed Ring Expansion of Aziridines with *N*-Sulfonyl-1,2,3-Triazoles. J. Org. Chem..

[49] Viudes O., Guarnieri-Ibáñez A., Besnard C., Lacour J. (2023). Regiodivergent Synthesis of Oxadiazocines via Dirhodium-Catalyzed Reactivity of Oxazolidines and α-Imino Carbenes. Synlett.

[50] Guarnieri-Ibáñez A., Medina F., Besnard C., Kidd S.L., Spring D.R., Lacour J. (2017). Diversity-oriented synthesis of heterocycles and macrocycles by controlled reactions of oxetanes with α-iminocarbenes. Chem. Sci..

[51] Ko Y.O., Jeon H.J., Jung D.J., Kim U.B., Lee S.-G. (2016). Rh(II)/Mg(O*^t^*Bu)_2_-Catalyzed Tandem One-Pot Synthesis of 1,4-Oxazepines and 1,4-Oxazines from *N*-Sulfonyl-1,2,3-triazoles and Glycidols. Org. Lett..

[52] Medina F., Besnard C., Lacour J. (2014). One-Step Synthesis of Nitrogen-Containing Medium-Sized Rings via α-Imino Diazo Intermediates. Org. Lett..

[53] Pospech J., Ferraccioli R., Neumann H., Beller M. (2015). Rhodium(II)-Catalyzed Annulation of Azavinyl Carbenes Through Ring-Expansion of 1,3,5-Trioxane: Rapid Access to NineMembered 1,3,5,7-Trioxazonines. Chem. Asian J..

[54] Lee K.R., Ahn S., Lee S.-G. (2021). Synergistic Pd(0)/Rh(II) Dual Catalytic [6 + 3] Dipolar Cycloaddition for the Synthesis of Monocyclic Nine-Membered *N,O*-Heterocycles and Their Alder-ene Rearrangement to Fused Bicyclic Compounds. Org. Lett..

[55] Li R., Li B., Zhang H., Ju C.-W., Qin Y., Xue X.-S., Zhao D. (2021). A ring expansion strategy towards diverse azaheterocycles. Nat. Chem..

[56] Du J., Yang X., Wang X., An Q., He X., Pan H., Zuo Z. (2021). Photocatalytic Aerobic Oxidative Ring Expansion of Cyclic Ketones to Macrolactones by Cerium and Cyanoanthracene Catalysis. Angew. Chem. Int. Ed..

[57] Ye J. (2021). Macrolactones via Photoinduced Ring Expansion of Cyclic Ketones. Chin. J. Org. Chem..

[58] Wang H., Shao H., Das A., Dutta S., Chan H.T., Daniliuc C., Houk K.N., Glorius F. (2023). Dearomative ring expansion of thiophenes by bicyclobutane insertion. Science.

